# The impact of detailed history taking: a case report of pediatric pulmonary and hepatic hydatid disease from Ecuador

**DOI:** 10.3389/fped.2024.1457463

**Published:** 2024-08-27

**Authors:** Camila Gallegos, Ariel Vargas, David Estrella, Alejandra Torres, Carlos Andrade, Yazmina del Carmen Lascano, Daniel Garzon-Chavez, Ana Cristina Aguilar

**Affiliations:** ^1^ School of Medicine, Universidad San Francisco de Quito (USFQ), Quito, Ecuador; ^2^ School of Medicine, University of the Americas (UDLA), Quito, Ecuador; ^3^ Postgraduate Medical Education, Pontificia Universidad Católica del Ecuador (PUCE), Quito, Ecuador; ^4^ Department of Pediatric Pulmonology, Hospital Carlos Andrade Marin (HCAM), Quito, Ecuador

**Keywords:** echinococcosis, hydatid disease, zoonotic transmission, pediatric infection, pulmonary and hepatic infection

## Abstract

**Background:**

Echinococcosis is a uncommon condition in pediatric patients, and encompasses alveolar and cystic forms, predominantly affecting the lungs and liver. Transmission occurs primarily through zoonotic means, such as the contamination of water and food by infected dog and other canid feces. Diagnosis can be challenging due to nonspecific symptoms that often mimic pneumonia.

**The case:**

A 6-year-old female patient from a rural area in Ecuador who initially presented with nonspecific symptoms indicative of pneumonia. However, further investigation into socio-environmental factors led to a diagnosis of pulmonary and hepatic hydatid disease.

**Conclusion:**

The timely and accurate diagnosis of this infectious disease enabled the patient to receive appropriate treatment and surgical intervention, leading to her complete recovery.

## Key points

• Echinococcosis is a parasitic infection primarily transmitted through zoonotic means.
• Hydatid disease is particularly rare in the pediatric population due to its pathophysiologic course.
• Diagnosis of echinococcosis can be particularly challenging due to its nonspecific symptoms.

## Introduction

Hydatid disease is caused by *Echinococcus* tapeworms, within this genus, four species hold significance: *E. granulosus*, *E. multilocularis*, and the less common *E. vogeli* (exclusively distributed in South America), and *E. oligarthrus* (1, 2). *E. granulosus'* adult form lives in the intestine of canids, the definite hosts; for the larvae state, the intermediate host includes sheep, cows and other herbivores. Humans become intermediate hosts through contact with infected dogs or by eating/drinking sources contaminated with canid feces (3, 4).

Once the eggs are ingested by humans, they hatch in the small intestine and release oncospheres, which penetrate the intestinal wall and travel through the lymphatic or blood vessels to the liver, lungs, and other organs, where they develop into hydatid cysts. Initially asymptomatic, the condition manifests symptoms as the cysts grow and pressure surrounding tissues (5).

This zoonosis correlates with livestock production and poor sanitary practices, especially in low socio-economic regions, with South American countries facing a significant burden. Ecuador reports low prevalence rates in the region, in 2022 a total of 18 hospital discharges were reported across the country (6, 7). Although cystic echinococcosis has generally been regarded as a disease that predominantly affects adults between ages of 20 and 59, it can also occur in early childhood, even in patients younger than 5 years old (8–10). Diagnosis typically involves imaging and serological studies (7). Treatment options include percutaneous cyst drainage, surgical intervention as the preferred treatment, and/or anti-parasitic drugs (11, 12).

## Case description

A 6-year-old female from Ecuador with a history of asthma and allergic rhinitis, presented to the emergency department with a 3-day history of abdominal pain, cough, fever, and vomiting on 5 occasions. Physical examination revealed a temperature of 38°C, desaturation (82%), diminished breath sounds and crackles in both lung fields. Laboratory studies showed leukocytosis (16700 × 10^3/*μ*l), elevated CRP 64.9 mg/L and procalcitonin 3.68 ng/ml, and a negative SARS-CoV-2 antigen test. A chest x-ray (CXR) revealed a consolidation in the left lung field. She was admitted, diagnosed with acute bacterial pneumonia, and initiated on ceftriaxone (1 g IV every 12 h) and acetaminophen (315 mg IV every 6 h) therapy.

**Figure 1:**
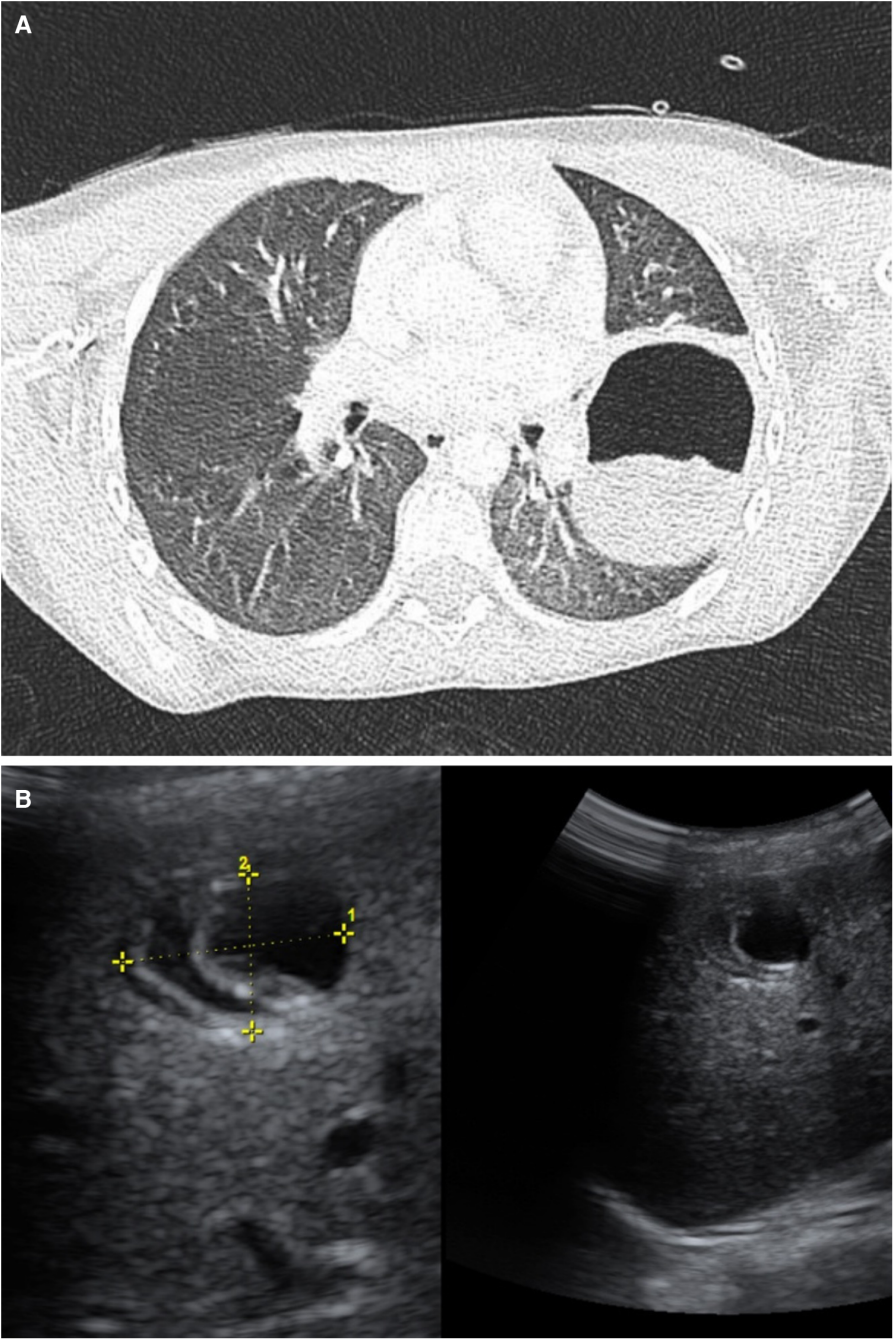
**(A)** Chest CT. Left lower lobe: A round, heterogeneous, hypodense image is denoted with a hyperdense halo, measuring 52 × 64 × 81 mm. Suggestive of a cyst. Bilateral diffuse ground glass opacity pattern observed in the lungs. **(B)** Abdominal Ultrasound: The liver is in a normal position with preserved echogenicity. A cyst-like lesion is observed in segment VIII, containing echogenic membranes, measuring 23 × 21 × 20 mm, and appearing avascular. Consistent with hydatid cyst.

On hospital day 1, she remained febrile with increased oxygen needs. Additionally, a rash on her legs developed, attributed to post-infusion urticaria. Consequently, it was decided to proceed with a chest CT (CCT), which revealed a cavitary lesion in the left inferior lobe, suggestive of complicated pneumonia with a lung abscess (Figure 1A). Given the presumed beta-lactam allergy presenting as urticaria and the patient's clinical deterioration, it was decided to switch the antibiotic therapy to levofloxacin (210 mg IV daily). Vancomycin (400 mg IV every 6 h) was initiated for MRSA coverage. Prednisone (20 mg orally in the morning) was also included.

Despite therapy, fever persisted, leukocytosis and inflammatory markers (IM) spiked. Due to the lack of improvement, the patient was transferred to a tertiary care hospital. Upon arrival, reevaluation revealed fever, productive cough, neutrophilia, and elevated IM. The previous CCT was reassessed, and an abdominal ultrasound revealed a cystic liver lesion (Figure 1B), suggesting hydatid disease. On further questioning about contact with animals, and drinking water and food sources, it was revealed that the patient had three unvetted household dogs.

Albendazole (170 mg orally every 12 h) was added to the ongoing antibiotic therapy due to the patient's hemodynamic status. A multidisciplinary approach was planned, and work-up for a possible left lower lobectomy was initiated. The subsequent ELISA test for *E. granulosus* using the SERION ELISA Classic Echinococcus IgG ESR107G was positive (20.28 U/ml). A definitive diagnosis of pulmonary CE was made, revealing that the patient did not have isolated acute bacterial pneumonia nor was it associated with the current infectious process.

A left anterolateral thoracotomy for left superior lobectomy was performed, including adhesiolysis, pleural lavage, and drainage. A 6 cm hydatid cyst was successfully resected without complications, while the small hepatic cyst was managed conservatively. Lung histopathological testing confirmed cystic echinococcosis (CE), while KOH was negative. The patient was stable when transferred to the Pediatric Intensive Care Unit with a left-sided chest tube (drained approximately 80 ml of hematic fluid) and requiring only oxygen by nasal cannula. Pain was controlled with acetaminophen (325 ml IV every 6 h), ketorolac (10 ml IV every 8 h), and morphine (20 ml mixed with saline 0.9% IV at 20 micrograms/kg/hour).

Two days later, the patient was transferred to a standard room tolerating room air oxygen. Systemic opioids were discontinued due to improved pain control. Albendazole therapy was maintained throughout the postoperative period, antibiotics were discontinued after 10 days of therapy. The patient remained stable, afebrile, and with normal lung sounds. She was discharged five days later with normal complete blood count (CBC), complete metabolic panel (CMP), liver function tests (LFT), and renal function tests (RFT).

Follow-up one month later with Pediatric Infectious Diseases was scheduled, it was recommended to obtain CXRs for household members and administer antiparasitic medication to pets. After six months, the patient completed antiparasitic therapy and achieved full recovery.

## Discussion

**Figure 2:**
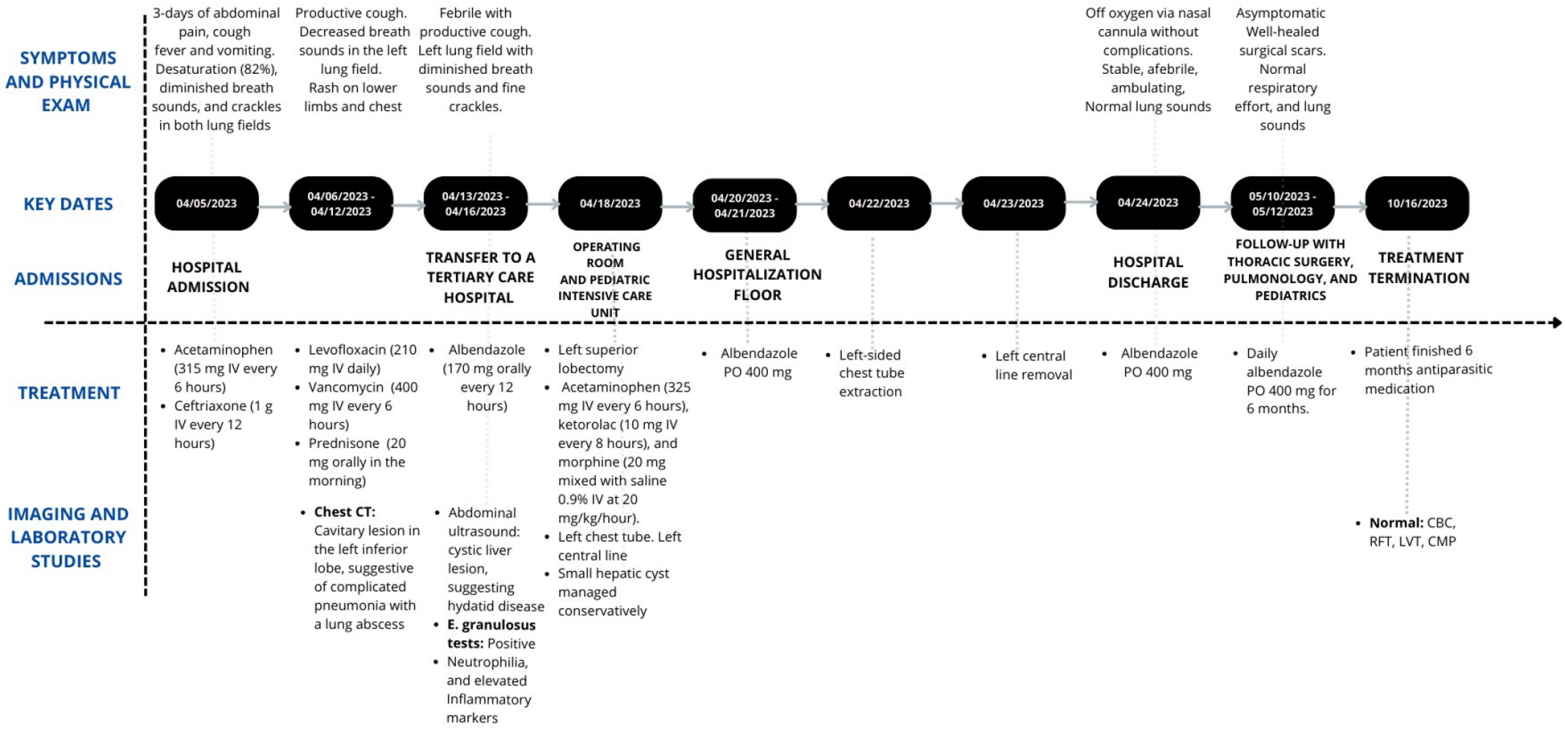
Timeline of key events in the case report.

This is an uncommon case of a child with pulmonary and hepatic hydatid disease, initially diagnosed as complicated pneumonia, leading to treatment with multiple antibiotic regimens and various challenges faced before being switched to the appropriate management (Figure 2). The clinical presentation and variants of echinococcosis have been extensively described. In the case of CE, it is associated with an indolent clinical course with most primary infections consisting of a single cyst, leading to damage and eventual organ dysfunction, especially of the lung–the most common site in the pediatric population, as seen in our patient–and liver (13–15). As cystic lesions remain asymptomatic for many years for the majority of patients or show a slow growth rate over decades, children constitute a minority of cases (8, 13).

When affected, pediatric patients can remain asymptomatic, even with large cystic lesions, due to their immature immune system and more elastic lung parenchyma compared to adults (16). However, this increased elasticity has been described as facilitating rapid growth of cysts in some patients (17). The clinical presentation will depend on the cyst's location, size, and their interaction with adjacent structures (18). Our patient presented with abdominal pain, cough, and vomiting, with physical examination finding desaturation, fever, and altered breath sounds, a presentation consistent with the available literature for this population (16). Only 10%–20% of cases are diagnosed during childhood, with most of the hydatid disease cases presenting in adulthood having been infected during this period (19).

CXR can be useful in narrowing the differential diagnosis by the presence of pathognomonic signs of CE such as floating membranes and/or the presence of a rounded uniform mass, none of which were present in our patient (16). Since no evident risk factors for CE had been identified, these findings led to a diagnosis of acute bacterial pneumonia. Eosinophilia was not present, it is a nonspecific finding present in less than 15% of patients, usually in those with ruptured cysts. Also, this patient was part of the allergic spectrum, making it less reliable (1, 8).

The patient was started on appropriate empiric antibiotic therapy with ceftriaxone for acute bacterial pneumonia per current IDSA guidelines but failed to respond and showed clinical deterioration (20, 21). CCT revealed a large cavitary lesion in the left inferior lobe, suggestive of complicated pneumonia with a lung abscess. Antibiotic therapy was switched to levofloxacin, due to allergic reaction to ceftriaxone, and vancomycin for empiric MRSA coverage following standard-of-care management and weighting risks and benefits of quinolone use in pediatric patients (20).

Patients with high-risk factors for CE, such as this patient, in whom further questioning uncovered exposure to unvetted dogs, the diagnosis is confirmed with a combination of imaging and serology studies, with ultrasound being the technique of choice (3). The differential diagnosis for CE is broad, as children typically present with non-specific respiratory symptoms that may suggest a more prevalent etiology (15). This includes abscess, empyema, congenital cystic abnormalities, etc.

This posed a challenging scenario, because unlike extrapulmonary cysts, intrapulmonary cysts do not calcify, and formation of daughter cysts is rare. They are more prone to local complications like secondary infection, rupture, host tissue reactions, etc (1). The location of pulmonary cysts has mixed evidence. Some authors describing the right pulmonary lobe as more common, presumably due to increased blood flow, which is contrary to the findings in our patient, while others find cysts more commonly located in the left lower lobes (15, 22).

By this point, with concordant epidemiological and clinical history, along with positive imaging and serology, a definite diagnosis of CE was established (13). A combined therapeutic approach was decided upon because current guidelines by the WHO recommend surgery and antiparasitic therapy with albendazole for cysts that are located in the lungs or those that are infected, both of which were present (8, 23, 24). The surgical technique depends on the location and size of the cysts. In this case, the patient's clinical scenario and cyst infection necessitated a left superior lobectomy (8, 18, 23, 25).

Albendazole therapy was started before the surgery following the guidelines dosing for CE (24). The use of preoperative albendazole has conflicting evidence. It has been shown to soften cysts and reduce intracystic pressures, facilitating easier cyst removal and reducing secondary hydatid disease and recurrence. However, other studies have shown that it can weaken the cyst walls, potentially causing ruptures and complications (8, 23). The size and appearance of the hepatic lesion, as it was not consistent with active disease, in this patient required only observation (26). The patient was followed for 6 months and stayed on albendazole therapy during this period, as standard-of-care, with no disease recurrence or adverse reactions caused by medication indicated by normal LFT and CBC (1, 8, 24). The patient and caregivers were educated on the etiology of the disease, its benign course, and measures for the prevention of recurrence. They expressed feeling relieved after the diagnosis was established, having spent days in the hospital receiving antibiotics without clinical improvement.

We employed a step-by-step approach, justifying each action taken in the care of this patient with relevant literature. By detailing the challenges encountered we are contributing to the knowledge in the management of pediatric infectious diseases presenting with non-specific symptomatology. The limitations encountered in this study include that our patient presented in one of multiple ways CE has been described in the literature, and this case may not represent the generalized clinical course of CE in the broader patient population. Additionally, the patient was treated in a resource-limited setting in a developing country, with management following current clinical practice guidelines, which may differ from practices in other settings.

## Conclusion

CE due to *E. granulosus* is a significant public health issue in developing regions, like South America. This case highlights the complexities of diagnosing and managing pulmonary CE in pediatric patients with nonspecific symptoms and no clear risk factors. In the absence of a suggestive epidemiological and clinical scenario, timely diagnosis becomes challenging. The patient did not respond to standard-of-care for acute bacterial pneumonia, prompting the need to consider other diagnoses. Reevaluation of her previous scans and an in-depth clinical history upon transfer revealed that the patient did indeed have risk factors for CE. Her diagnosis was confirmed by ultrasound imaging in combination with serologic and histopathologic testing, allowing for adequate management and clinical improvement. Prompt and accurate diagnosis of parasitic infections is key for antibiotic stewardship, as it prevents the misuse of antibiotics, which can cause side effects and promote bacterial resistance.

## Data Availability

The original contributions presented in the study are included in the article/Supplementary Material, further inquiries can be directed to the corresponding author.
